# First Direct Evidence of Pan-African Orogeny Associated with Gondwana Assembly in the Cathaysia Block of Southern China

**DOI:** 10.1038/s41598-017-00950-x

**Published:** 2017-04-11

**Authors:** Longming Li, Shoufa Lin, Guangfu Xing, Yang Jiang, Jian He

**Affiliations:** 1 School of Resources and Environment, Hefei University of Technology, Hefei, 230026 P.R. China; 2 Department of Earth and Environmental Sciences, University of Waterloo, 200 University Avenue West, Waterloo, Ontario N2L 3G1 Canada; 3 Nanjing Institute of Geology and Mineral Resources, Nanjing, 210016 P.R. China

## Abstract

Metamorphic zircon from a hornblendite in the South China Block (SCB) yield U-Pb age of 533 ± 7 Ma and Hf model ages from 900 to 1200 Ma. Geochemical and isotopic characteristics indicate that primary magma of the hornblendites was probably derived from an enriched asthenospheric mantle source. This Late Neoproterozoic–Cambrian (Pan-African) metamorphic event provides the first direct evidence that the SCB was an integral part of the Gondwana assembly. Combined with available geological data which show that the SCB has great affinity with India or Australia, the Pan-African metamorphic event most likely belongs to the eastern Kuunga orogeny. We propose that the SCB was located at the nexus between India, Antarctica and Australia along the northern margin of East Gondwana, with the Cathaysia Block connecting western Australia whereas the Yangtze Block facing northern India at *ca*. 533 Ma.

## Introduction

The history of Gondwana supercontinent and its configuration have been the focus of many investigations^1–5^. The Late Neoproterozoic–Cambrian (Pan-African) orogeny, which can be regarded as a diagnostic feature for reconstructing the Gondwana supercontinent, has been recognized in many Gondwana continental fragments including India, East Antarctica and Africa^6–9^. Although Pan-African-aged detrital or inherited/xenocrystic zircons have been found in some Paleozoic to Mesozoic sedimentary or igneous rocks of the South China Block (SCB)^10–13^, unequivocal Pan-African magmatic or metamorphic rocks have not been identified in the SCB. Thus, most workers believe that the Gondwana-forming orogeny did not affect the SCB, and whether the SCB was an integral part of the Gondwana supercontinent and, if so, where it was located in Gondwana are still uncertain.

In this article, we report the first evidence for a significant Pan-African metamorphic event in the SCB. The results provide robust constraints on the location of the SCB in the Gondwana supercontinent.

## Geological setting and sample description

**Figure 1:**
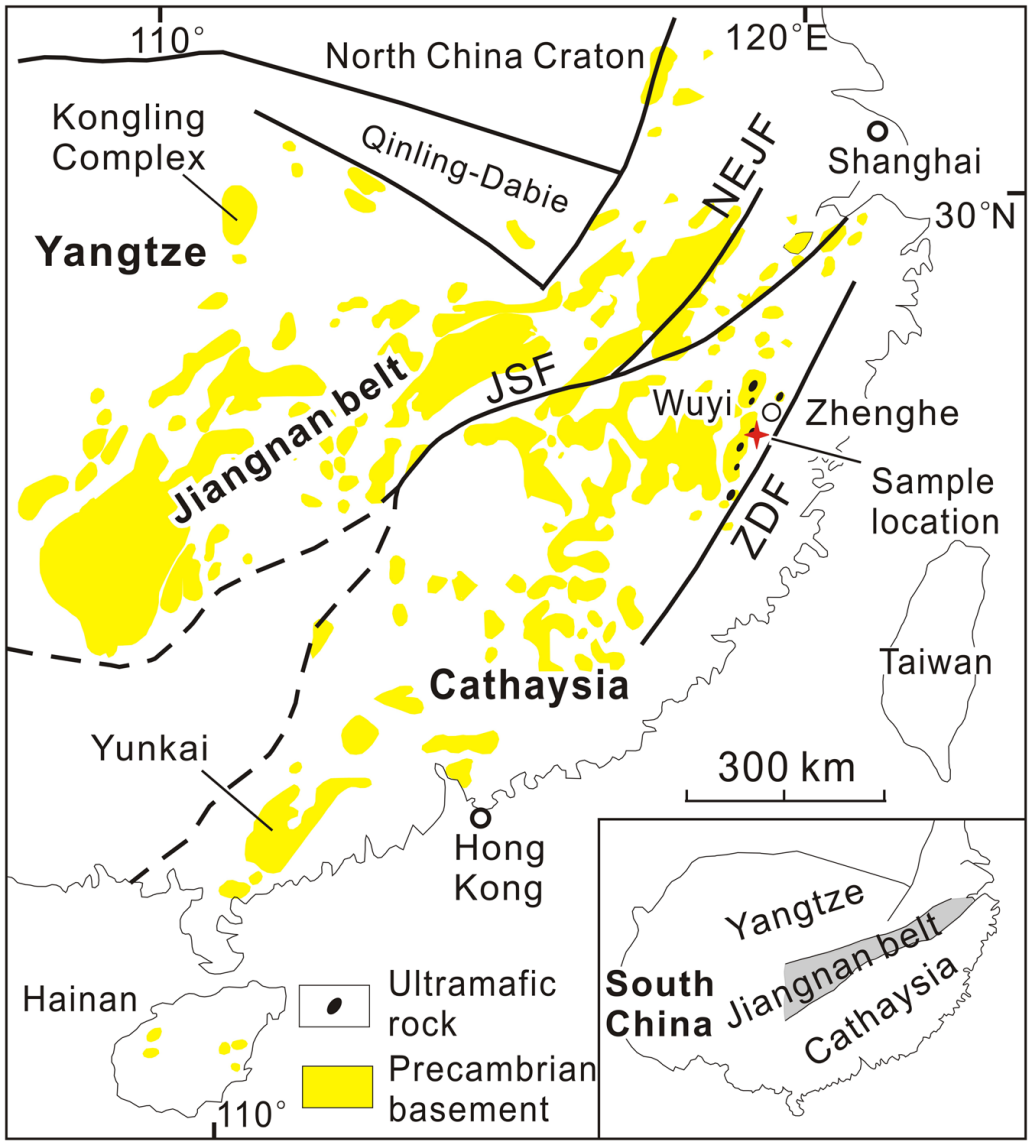
Simplified geological map of eastern South China (modified from Zhao and Cawood^52^), showing the location of the honblendites.

The SCB is composed of the Yangtze Block to the northwest and the Cathaysia Block to the southeast. The intervening Jiangnan orogen considered as a collisional belt^14–17^ (Fig. 1) between the two, with the proposed timing of collision varying from late Mesoproterozoic^14^ to middle Neoproterozoic^15–17^.

The Yangtze Block is characterized by an Archean-Paleoproterozoic crystalline basement surrounded by late Mesoproterozoic to early Neoproterzoic folded belts, unconformably overlain by unmetamorphosed late Neoproterozoic and younger covers^18, 19^. The Cathaysia Block mainly consists of Paleoproterozoic and Neoproterozoic rocks^20, 21^. Predominant Precambrian basement of the Cathaysia Block is bounded by the Jiangshan-Shaoxing Fault (JSF) to the northewest and the Zhenghe-Dapu Fault (ZDF) to the southeast (Fig. 1). It includes metasedimentary and metavolcanic rocks that have been metamorphosed from upper greenschist to granulite facies, in the early Palaeozoic^22, 23^, and at least partly also during the Pan-African orogeny (see below).

**Figure 2:**
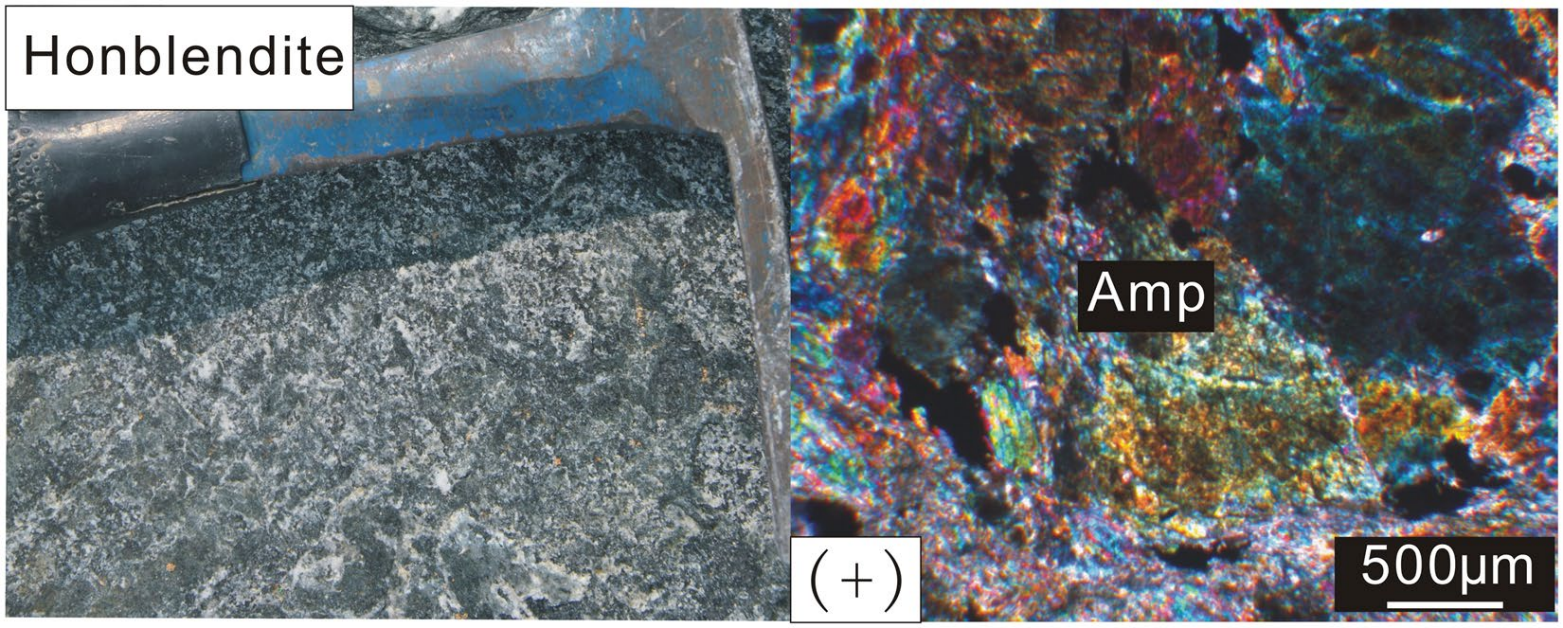
Field and microscope photographs for the hornblendites of the Cathaysia Block.

Mafic-ultramafic rocks are exposed as lenses in the Precambrian basement of the Cathaysia Block. The ultramafic rocks include serpentinized peridotite, clinopyroxenite and hornblendite. They are commonly associated with meta-gabbros and basalts of ca. 830–860 Ma^24^. The ultramafic lenses are hosted in gneisses and mica schists of Neoproterozoic age along the ZDF. They are flattened parallel to the regional metamorphic foliation and thus likely experienced the same deformation as their host rocks^24^. This study is mainly concerned with the hornblendites exposed in the Shitun village, Zhenghe County, South China (Fig. 1). The hornblendites mainly consist of >95% hornblende that is characterized by anhedral crystal habits. There are small amount of plagioclase, quartz, titanite and opaque minerals. Grain boundaries vary from straight to variably indented (Fig. 2).

## Results

### Zircon U-Pb geochronology and Hf isotopic study

Zircons from a hornblendite sample (14WY-8-15, N27°20′25.6″, E118°45′14.3″) are mostly rounded to subhedral, transparent and light brown in color. Crystal sizes range from 50 to 150 μm, with length/width ratios ranging from 3:2 to 1:1.

**Figure 3:**
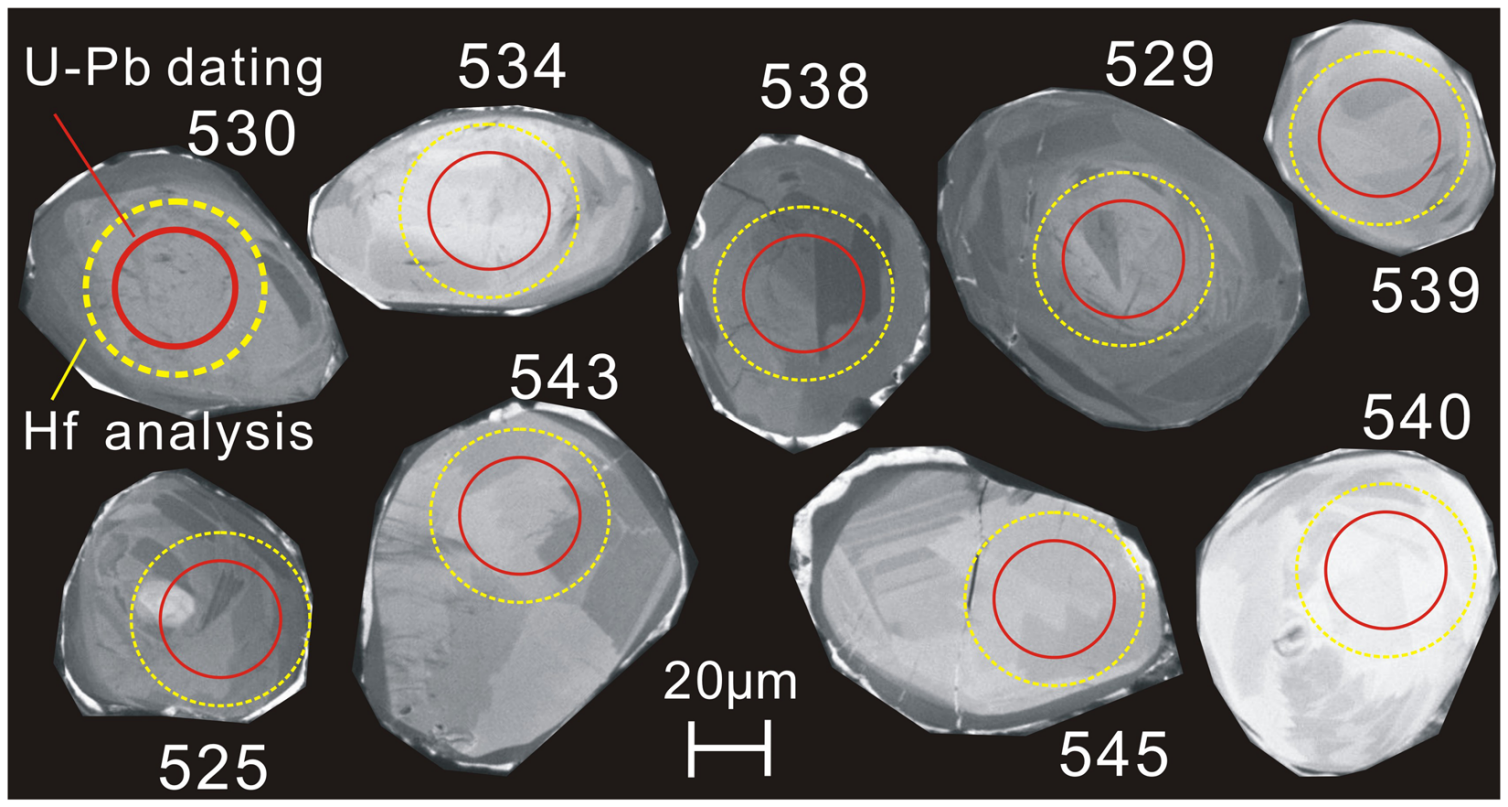
Representative CL images of analyzed zircons from the hornblendite.

The zircons are interpreted to be metamorphic in origin based on the following: (1) CL images reveal sector zoning that is typical of metamorphic zircons (Fig. 3); (2) The zircons are abundant in the sample, whereas igneous zircons should be rare, if any, in ultramafic igneous rocks; and (3) the hornblendites of this study are demonstrated to be metamorphic rocks (see below). As discussed below, during high-grade metamorphism, zircon can grow in hornblende-rich rocks.

**Figure 4:**
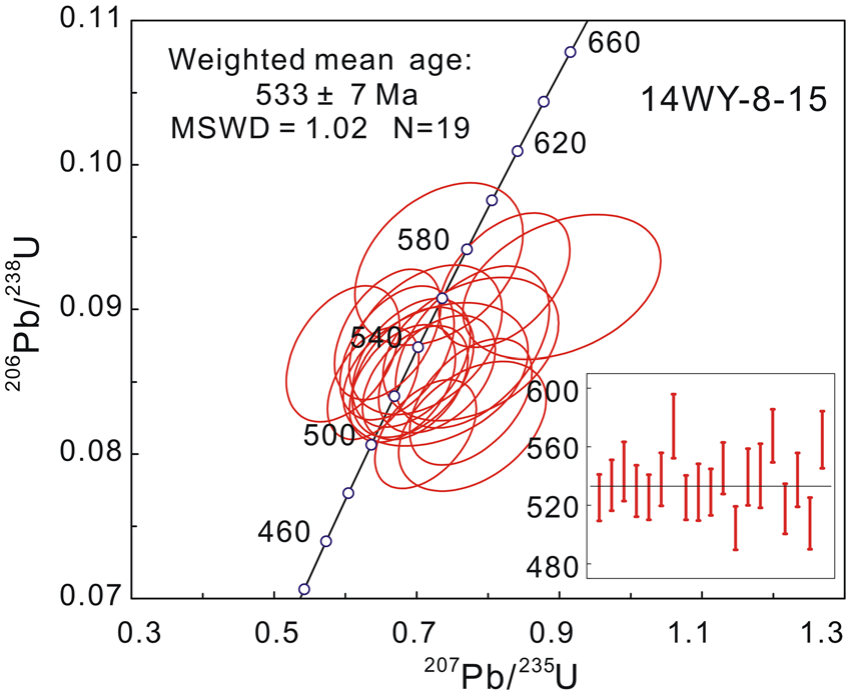
Concordia diagram of zircon U-Pb data for the dated hornblendite.

A total of nineteen zircon grains were analyzed. U-Pb results are given in Table 1. More details (including Hf data discussed below) are given in Supplementary Table S1. The data are all concordant or nearly concordant and give a weighted mean ^206^Pb/^238^U age of 533 ± 7 Ma (MSWD = 1.02, n = 19) (Fig. 4). This age is regarded as the metamorphic age of the hornblendite. Th/U ratios dominantly range from 0.32 to 0.74.

**Table 1:** LA-ICP-MS U-Pb data for the hornblendite (14WY-8-15) from Shitun village, Zhenghe County, South China.

Grain	U	Th/U	Ratios (common-Pb corrected)					Ages (common- Pb corrected, Ma)						
			^207^Pb/^235^U	1σ	^206^Pb/^238^U	1σ	Err*	^207^Pb/^206^Pb	±1σ	^207^Pb/^235^U	±1σ	^206^Pb/^238^U	±1σ	%Disc
1	97.1	0.35	0.6800	0.0491	0.0849	0.0027	0.4362	600	159	527	29.7	525	15.9	99%
2	80.5	0.39	0.6925	0.0546	0.0863	0.0029	0.4304	656	184	534	32.7	534	17.4	99%
3	34.5	0.37	0.7125	0.0708	0.0879	0.0034	0.3902	643	233	546	42.0	543	20.2	99%
4	55.7	0.52	0.6947	0.0563	0.0857	0.0030	0.4271	654	186	536	33.7	530	17.6	98%
5	103	0.43	0.6979	0.0489	0.0849	0.0026	0.4362	561	165	538	29.2	525	15.4	97%
6	55.3	0.35	0.6725	0.0528	0.0870	0.0031	0.4463	457	185	522	32.1	538	18.1	97%
7	22.9	0.42	0.7312	0.0778	0.0931	0.0037	0.3744	546	233	557	45.6	574	21.9	97%
8	105	0.74	0.6388	0.0422	0.0849	0.0026	0.4549	391	163	502	26.2	525	15.2	95%
9	47.9	0.32	0.7265	0.0855	0.0855	0.0033	0.3239	831	264	555	50.3	529	19.4	95%
10	89.9	0.44	0.7283	0.0540	0.0855	0.0027	0.4213	683	178	556	31.8	529	15.9	95%
11	96.5	0.37	0.6592	0.0492	0.0883	0.0030	0.4507	467	181	514	30.1	545	17.6	94%
12	92.5	0.37	0.7125	0.0469	0.0814	0.0025	0.4641	720	152	546	27.8	504	14.8	92%
13	24.6	0.38	0.7759	0.0813	0.0873	0.0033	0.3575	955	256	583	46.5	539	19.4	92%
14	25.7	0.43	0.7781	0.0712	0.0874	0.0037	0.4617	991	231	584	40.7	540	21.9	92%
15	69.5	0.40	0.8227	0.0606	0.0920	0.0031	0.4530	831	167	610	33.8	567	18.1	92%
16	71.4	0.42	0.7758	0.0567	0.0836	0.0029	0.4711	857	169	583	32.4	518	17.1	88%
17	43.6	0.47	0.5965	0.0525	0.0869	0.0031	0.4054	235	207	475	33.4	537	18.4	87%
18	53.5	0.41	0.7781	0.0683	0.0819	0.0030	0.4120	1039	207	584	39.0	508	17.7	85%
19	43.6	0.40	0.9034	0.0915	0.0916	0.0033	0.3558	989	222	654	48.8	565	19.5	85%

The (^176^Hf/^177^Hf)_i_ values are concentrated in a range of 0.282386–0.282572. Their single-stage model ages (*T*_DM1_) are of 0.94–1.2 Ga. Zircons from mafic rocks from the same area also have *T*_DM1_ of 0.9–1.4 Ga^24^. These features reflect that the principal magma of these mafic-ultramafic rocks was derived from a mantle source with late Mesoproterozoic to early Neoproterozoic model ages. It is noted that morphology and *T*_DM1_ age (~1.0 Ga) of the ~530 Ma detrital zircons^10^ from the Cathaysia Block are similar to that of the zircons of this study, suggesting that some of the detrital zircons might have been sourced from the hornblendites.

### Geochemical features of the hornblendites

Major and trace elements results are given in the Supplementary Table S2. The hornblendites are characterized by relatively low SiO_2_ (43.8–44 wt.%) and high MgO (9.99–10.5 wt.%), FeO^T^ (14.9–15.4 wt.%) and CaO (12–13.4 wt.%) contents with high Mg^#^ (57.2–58.5) and CaO/Al_2_O_3_ ratios (0.96–1.15). They have high TiO_2_ (3.05–3.39 wt.%) and moderate total alkaline contents (2.05–2.6 wt.%) with Na_2_O (1.66–2.03 wt.%) higher than K_2_O (0.33–0.66) contents. The hornblendites also have high compatible element contents (Cr 381–430 ppm, Co 54–56 ppm and Ni 175–197 ppm). Their moderate alkali and high TiO_2_ contents denote a tholeiitic affinity of their primary magma. On the chondrite-normalized REE diagrams (Fig. DR1) they exhibit fractionated LREE patterns (La/Yb_N_ = 4.97–5.34) with weak negative to slightly positive Eu anomalies (Eu/Eu* = 0.95–1.21). Their primitive mantle-normalized diagrams are characterized by moderate enrichments in most trace elements, such as Zr (195–231 ppm), Ta (0.93–1.19), Nb (14.1–17.2 ppm) and Th (0.94–2.57 ppm), with slight depletion to enrichment in Sr (286–895 ppm) (Fig. DR2). Such trace element patterns without significant Nb–Ta depletion relative to La are typical of intraplate tholeiitic basaltic rocks.

Sr-Nd and Re-Os isotopic results are listed in Supplementary Tables S3 and S4. They show high initial ^87^Sr/^86^Sr ratios of 0.7081 to 0.7095 and relatively consistent ^143^Nd/^144^Nd ratios of 0.512296 to 0.512304. The initial Sr-Nd isotope compositions of the hornblendites are consistent with those from enriched mantle source. The hornblendites have high Re (475–2669 ppt) and Os (293–454 ppt) concentrations. The measured ^187^Re/^188^Os and ^187^Os/^188^Os ratios range from 7.62 to 42.9 and 0.1831 to 0.4748, respectively. In the plot of Re/Os and Os, they fall into the field of mantle melt, indicating that the hornblendites are not mantle residues (Fig. DR3). In the plot of Os and ^187^Re/^188^Os, they fall into the field of OIB (Fig. DR4).

## Discussion

Since igneous zircons are absent in the hornblendites, the timing of the emplacement of the ultramafic magma cannot be directly determined. A previous study shows that the rhyolites of the bimodal volcanic rocks from the Mamianshan Group, of which the hornblendites of this study form a part, has yielded a U-Pb zircon age of 818 ± 9 Ma^25^. In addition, mafic rocks in the same area yield weighted mean SHRIMP ^206^Pb/^238^U age of 830–860Ma^24^. The ultramafic rocks of this study are spatially associated with the mafic rocks and are thus likely also of a Neoproterozoic age, which is consistent with their Hf model ages.

The geochemical features of the hornblendites are characterized by enrichment of high field strength elements such as TiO_2_, Zr, Ta and Th. Overall OIB-like trace element patterns, without positive Pb and Sr anomalies or negative Nb-Ta anomalies, suggest that they are unlikely to have formed from partial melting of metasomatized lithospheric mantle. Since the TiO_2_ content of basaltic magma from asthenosphere mantle sources is relatively high compared to magmas from lithospheric mantle sources^26^, the relatively high contents of TiO_2_ (3.05–3.39 wt.%), Zr (195–231 ppm) and Zr/Sm ratios (21.1–23.3) in the hornblendites indicate that the ultramafic magma were probably derived from an asthenosphere mantle reservoir. Re-Os isotopic data also shows that the primary magma of the hornblendites have sources similar to OIB. The slightly enriched Sr-Nd isotopic compositions further suggest that the ultramafic magma was from enriched asthenosphere mantle source.

High Sc (38.7–44.2 ppm) and V (425–466 ppm) concentrations in the ultramafic rocks are consistent with an igneous origin^27^. These samples have relatively low Mg# (56–58) and Ni (175–197 ppm), reflective of certain degree of the fractionation crystallization of the olivine and clinopyroxene. In the discrimination diagrams (Fig. DR5–6), these samples plot in the field of within-plate basalt or within-plate tholeiite, similar to the coeval mafic rocks, suggesting that the mafic-ultramafic magma erupted in a continental rift environment which was presumably triggered by asthenospheric upwelling. Shu *et al*.^24^ suggest that the ultramafic rocks, pillow basalts, gabbros, dykes, or more generally the bimodal igneous rocks from the Mamianshan Group, were emplaced and partly accumulated in the same rift basin. The evolution of the rift basin in the Cathaysia Block is coeval with the Neoproterozoic basins in many parts of South China, which is similar to that of the Adelaide rift system in southeastern Australia that was related to the breakup of Rodinia supercontinent^28^.

**Figure 5:**
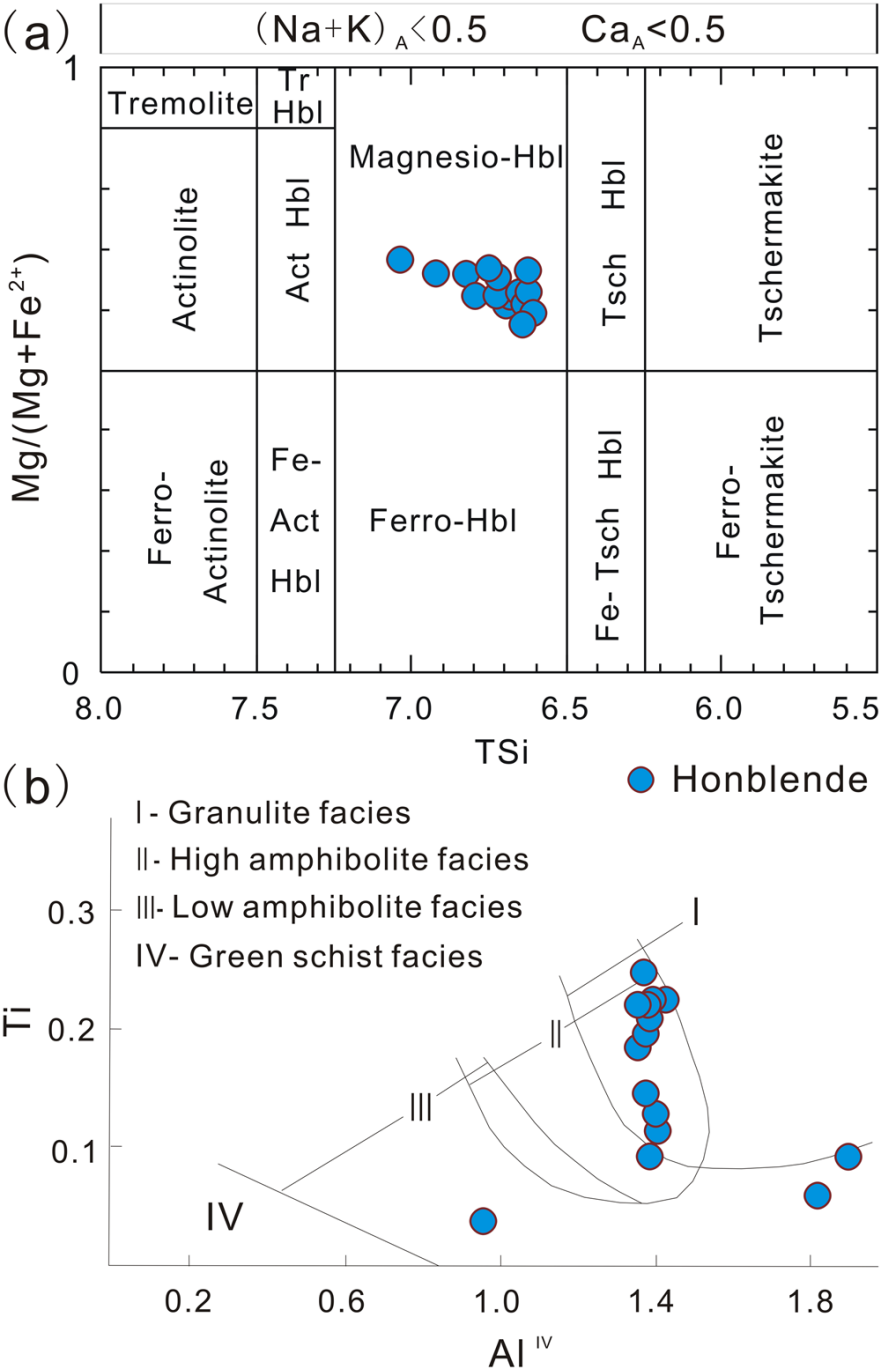
Amphibole chemistry from the hornblendite. (**a**) Si-Mg/Mg+Fe^2+^ (after Leak *et al*.^53^); (**b**) Al-Ti diagram (after Jin^54^).

There are two possible processes for the formation of the amphibole in the hornblendites. Firstly, they can result from the metasomatic alteration of pyroxene by metamorphic fluids in ultramafic rocks^29^. Secondly, they can originate as an igneous mineral but were recrystallized during high grade metamorphism^30^. The hornblendites in this study is composed of >95% amphibole. Petrographic observations and geochemical data suggest that amphibole in the hornblendites is magnesio-hornblende, a common metamorphic mineral (Supplementary Table S5, Fig. 5a). Since the temperature and pressure control the Ti and Al content of the amphibole, the high Ti and Al content indicate a higher temperature and pressure of the amphibolites. These amphiboles plot in the high amphibolite-granulite facies field (Fig. 5b). Thus, the hornblende appears to have originated as an igneous mineral but underwent extensive recrystallization during amphibolite to granulite facies metamorphism.

Ultramafic rocks are silica unsaturated, and common minerals such as zircon are typically absent. However, there are large amount of zircons in the hornblendites of this study. As described above, these zircons with sector zoning are typical metamorphic zircons, which most likely formed during high grade metamorphism. Since the Ti content of the zircon is correlated with the equilibration temperature, the formula log(Ti_zircon_) = 6.01 ± 0.03 − (5080 ± 30)/T can be used to calculate the metamorphic temperature^31^ (Supplementary Table S6 **)**. The temperature (~700 °C) is obviously lower than the crystallization temperature of the ultramafic magma, but is consistent with the above metamorphic temperature of the magnesio-hornblende. The crystallographic lattice of hornblende can accommodate Zr in significant amounts, thus Zr is a compatible element in hornblende. Simple calculations show that reaction of hornblende to form non-Zr bearing phases will release sufficient Zr to account for at least some new zircon growth^32^ and therefore hornblende can be regarded as a source for zirconium during high-grade metamorphism.

Various configurations and models have been proposed for the timing and tectonics of the assembly of the Gondwana supercontinent. Some workers argued that the SCB was an isolated continental block in the paleo-Pacific during the assembly of Gondwana^33^. However, it has been gradually accepted that the SCB was closely related to the Gondwana assembly in the Late Neoproterozoic to Early Paleozoic^34, 35^. The *ca*. 533 Ma metamorphic event documented in the hornblendite indicate that the SCB preserves the record of a major Pan-African orogeny, supporting that the SCB was an integral part of the Gondwana assembly. More significantly, it can help to constrain the location of the SCB in Gondwana.

Available geological data shows that the SCB has great affinity with India or Australia. However, the exact position of the SCB in Gondwana has not been well constrained. For instance, detrital zircon age patterns indicate that the SCB were either adjacent to northern India^13, 36^ or between India and Australia^10–12, 34^. Paleomagnetic data also allow the SCB being either near Eastern Australia or adjacent to Western Australia and India in late Neoproterozoic to Early Paleozoic^37–39^. Faunal affinities between the SCB and the India-Himalaya region appeared throughout much of the Early Paleozoic^40^. Comparable stratigraphic records between northern India and the Yangtze Block also exist in the Neoproterozoic to Early Paleozoic^41^.

**Figure 6:**
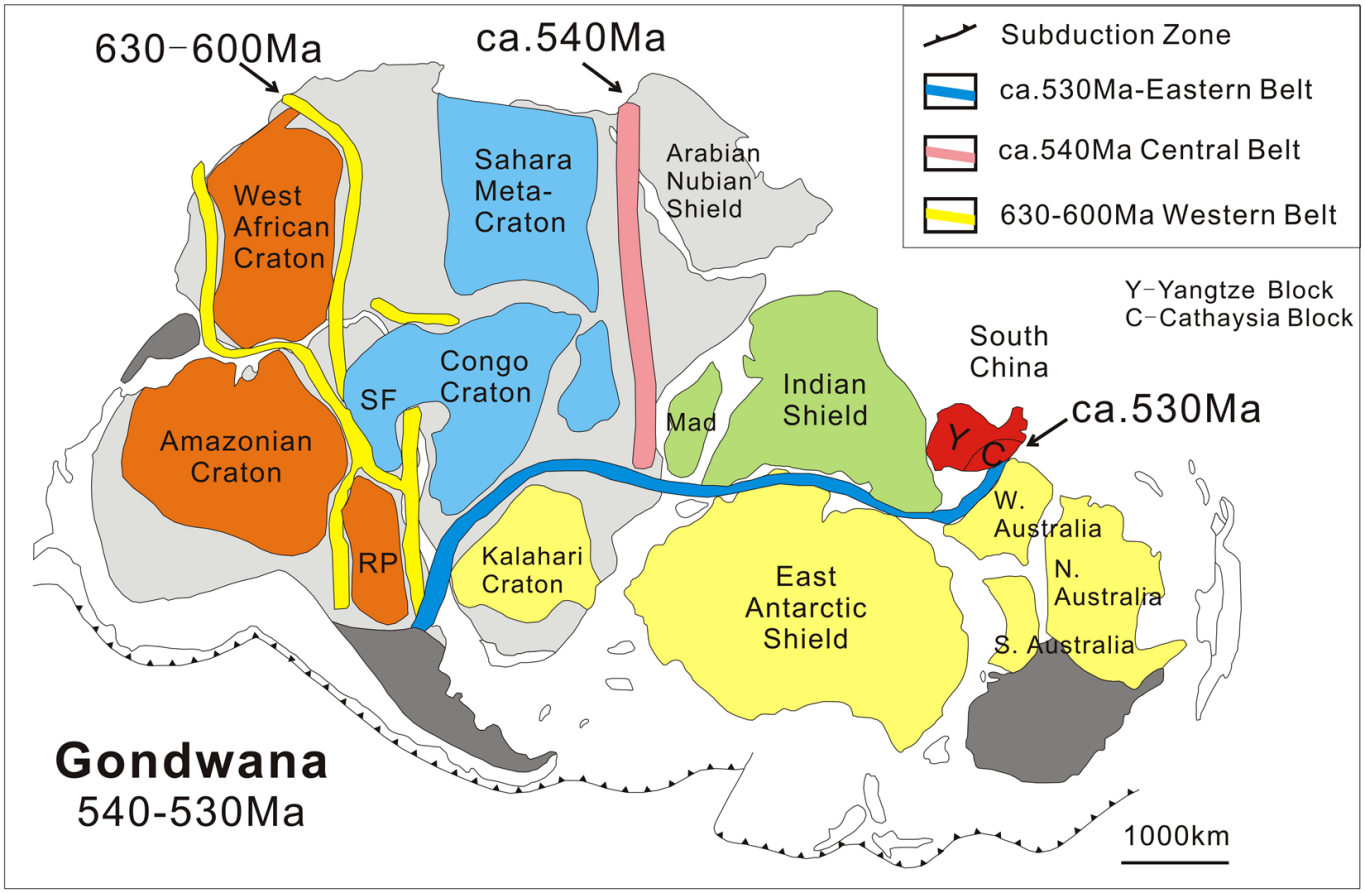
Gondwana reconstruction diagram showing the location of South China Block at 540–530 Ma (modified after Santosh *et al*.^9^).

The Pan-African orogenic belts in general are believed to have formed between 650–500 Ma. They are further classified into three belts, including the western belt (the Brasiliano-Damara orogen), the central belt (the East African Orogen or Mozambique orogen) and the eastern belt (the Kuunga orogen)^7^ (Fig. 6). The western belt was mostly consolidated at 630–600 Ma^9^. The N-S trending East African Orogen (the central belt), developed during the closure of the Mozambique Ocean, is also well-dated between 549–535 Ma^42^. The Kuunga orogen (the eastern belt) runs along the western margin of Australia, and presumably continues along northern Antarctica^43^. Parts of Sri Lanka and Madagascar may also belong to the eastern belt^44^. Meert^45^ interpreted that the Kuunga orogen was 570–530 Ma, coinciding with the closure of the Mozambique Ocean in the central belt, whereas Squire *et al*.^44^ suggested an age of 530–515 Ma. Combining with the above mentioned geological data, the *ca*. 533 Ma metamorphic event in the SCB most likely belongs to eastern Kuunga orogeny.

It should be noted that northern India was not involved in the above mentioned main period of Gondwana-forming orogeny^46^, although India forms the ‘heart’ of Gondwana^47^. Therefore, based on the occurrence of the Pan-African orogeny in the Cathaysia Block and that recognized in the western Australia and northern Antarctica, we believe that the Cathaysia Block was more likely connected to west Australia and East Antarctica than to north India at *ca*. 533 Ma. Also, considering the marked similarity between stratigraphic records in the Yangtze Block and those recognized in northern India, it is suggested that the Yangtze Block is adjacent to northern India. We thus present a modified paleogeographic reconstruction for the SCB in the Gondwana supercontinent (Fig. 6). During the Pan-African period (*ca*. 533 Ma), the SCB was likely located at the nexus between India, Antarctica and Australia, along the northern margin of East Gondwana, with the Cathaysia Block connecting western Australia whereas the Yangtze Block facing northern India.

## Conclusions

Metamorphic zircon from a hornblendite in the South China Block (SCB) yield U-Pb age of 533 ± 7 Ma and Hf model ages from 900 to 1200 Ma. The primary magma of the hornblendites was probably derived from an enriched asthenospheric mantle source triggered by asthenospheric upwelling. The *ca*. 533 Ma high-grade metamorphism recorded in the hornblendites provides first direct evidence for a major Pan-African orogeny in the SCB. Combined with available paleomagnetic data, faunal affinities and comparative stratigraphic records as well as comparative detrital zircon age patterns, the data indicate that the SCB was likely located at the nexus between India, Antarctica and Australia at *ca*. 533 Ma, with the Cathaysia Block connecting western Australia whereas the Yangtze Block facing northern India.

## Methods

### Zircon U-Pb geochronology and Hf isotopic analyses

Measurements of U, Th and Pb isotopes of zircon were conducted using an agilgent 7500a quadruple (Q)-ICPMS attached with a Geolas laser-ablation system equipped with a 193 nm Ar-F-excimer laser at the Hefei University of Technology (HFUT). Zircon U-Th-Pb ratios and absolute abundances were determined relative to the standard zircon 91500. Spot size in the range of 40–50 µm was used for data collection. The standard 91500 and GJ-1 zircons were used to calibrate the U-Th-Pb ratios and absolute U abundances. The instrumental setting and detailed analytical procedure have been described by Yuan *et al*.^48^. Uncertainties on single analyses are reported at the 1σ level; mean ages for pooled U-Pb analyses are quoted with a 95% confidence interval. Data reduction was carried out using the Isoplot/Ex 3 software^49^.

Zircon *in-situ* Hf isotopic analysis was carried out using a Geolas-193 laser-ablation microprobe at the Guangzhou Institute of Geochemistry (GIG), Chinese Academy of Sciences (CAS). External calibration was made by measuring zircon standard 91500 with the unknowns during the analyses to evaluate the reliability of the analytical data, which yielded a weighted mean ^176^Hf/^177^Hf ratio of 0.282307 ± 31 (2σ). This value is in good agreement with the recommended value of 0.282305 ± 12 (2σ). The mean β_Yb_ value was applied for the isobaric interference correction of ^176^Yb on ^176^Hf in the same spot. The ratio of ^176^Yb/^172^Yb (0.5887) was also applied for the Yb correction. Details of Hf isotopic analytical method followed Wu *et al*.^50^.

### Whole-rock analyses

Rock chips were ground in an agate mill and prepared for whole-rock analysis. Major element oxides were analyzed on fused glass disks with a Rigaku RIX 2000 X-ray fluorescence spectrometer (XRF) at GIG, CAS. Based on the measured values of rock standards (BHVO-1 and AGV-1), the analytical uncertainties are estimated to be better than 3% for all the major elements.

Trace elements were determined by Perkin-Elmer Sciex ELAN 6000 inductively coupled plasma mass spectrometry (ICP-MS) at GIG, CAS. Sample powders were decomposed in a mixture of distilled HF-HNO_3_ in Savillex Teflon beakers for 6 days at 120 °C. The sample solution was dried and the residue dissolved in 50 ml 1% HNO_3_ for ICP-MS analysis. A set of international standards including BHVO-1, G-2, GSR-3 and AGV-1 was used to estimate the accuracy and precision of the analyses.

Sr-Nd isotopic analyses were carried out at the Guiyang Institute of Geochemistry, Chinese Academy of Sciences. Sample powders (~100 mg) were dissolved in distilled HF-HNO_3_ in Savillex Screwtop Teflon beakers at 150 °C overnight. Sr and REE were separated on columns made of Sr and REE resins of the Eichrom Company using 0.1% HNO_3_ as eluant. Separation of Nd from the REE fractions was carried out on HDEHP columns with a 0.18N HCl as an eluant. Isotopic compositions were determined using a Micro Mass Isoprobe Multi-collector Mass Spectrometer (MC-ICP-MS). The mass fractionation corrections for Sr and Nd isotopic ratios are based on ^86^Sr/^88^Sr = 0.1194 and ^146^Nd/^144^ Nd = 0.7219, respectively. ^87^Rb/^86^Sr and ^147^Sm/^144^Nd ratios were calculated using the Rb, Sr, Sm and Nd abundances measured by ICP-MS. The measured ^87^Sr/^86^Sr ratio of the (NIST) SRM 987 standard and ^143^Nd/^144^Nd ratio of the La Jolla standard are 0.710265 ± 12 (2σ) and 0.511862 ± 10 (2σ), respectively.

Re-Os isotopic compositions were determined on the MC-ICPMS mass spectrometer at the State Key Laboratory of Continental Dynamics, Northwest University, Xi’an, China. The analytical procedure is described in detail in Li *et al*.^51^. Samples were spiked with solutions enriched in ^190^Os and ^185^Re and digested in reverse aqua regia (2:1 HNO_3_:HCl) in Carius tubes. Osmium was extracted by carbon tetrachloride solvent extraction and further purified by micro distillation. Rhenium was extracted and purified from the remaining solution by anion exchange using AG 1 · 8 resin (100–200 mesh). Instrumental mass fractionation for Os was corrected by normalizing the measured ^192^Os/^188^Os to 3.08271. Oxide corrections were made using ^17^O/^16^O = 0.00037 and ^18^O/^16^O = 0.002047. Rhenium isotopic abundances were determined after total evaporation of the samples on the filaments. This method eliminates the effect of instrumental mass fractionation and yields isotopic ratios more accurate than conventional NTIMS measurement techniques. Total procedural blanks were ~7 pg for Re, and ~2 pg for Os with ^187^Os/^188^Os of ~0.298. Contribution of the blank to measured Os concentrations and ^187^Os/^188^Os were <10% and <5% respectively. Precision of ^187^Os/^188^Os measurements was better than 0.4% (2σ).

### Major oxides of minerals

Chemical compositions of amphiboles of hornblendite were analyzed using the JOEL JXA8230 electron microprobe equipped at the School of Resources and Environment Engineering, HFUT, China. The analytical conditions were 15 kV accelerating voltage, a beam current of 20 nA with an electron beam size of 5 μm and 10–20 s counting time. Standards for this laboratory were natural and synthetic minerals.

## Electronic supplementary material

Supplementary information
