# Supplementary material for: First Direct Evidence of Pan-African Orogeny Associated with Gondwana Assembly in the Cathaysia Block of Southern China
Source: Sci Rep. 2017 Apr 11;7:794. doi: 10.1038/s41598-017-00950-x (PMC5429774; doi:10.1038/s41598-017-00950-x)
Supplement: Supplementary file 1 — Supplementary information [file 41598_2017_950_MOESM1_ESM.pdf]

First Direct Evidence of Pan-African Orogeny Associated with Gondwana Assembly in the Cathaysia Block of Southern China

Longming Li[a, *], Shoufa Lin[b, a], Guangfu Xing[c], Yang Jiang [c], Jian He[a]

[a]*School of Resources and Environment, Hefei University of Technology, Hefei 230026, PR China*

[b]*Department of Earth and Environmental Sciences, University of Waterloo, 200 University Avenue West, Waterloo, Ontario N2L 3G1, Canada*

[c]*Nanjing Institute of Geology and Mineral Resources, Nanjing 210016, PR China*

Table S1. Zircon Lu-Hf isotopic data for the hornblendite (14WY-8-15) from Shitun village, Zhenghe County, South China.

Analysis	Age(Ma)	$^{176}Hf/^{177}Hf$	2σ	$^{176}Yb/^{177}Hf$	2σ	$^{176}Lu/^{177}Hf$	2σ	$^{176}Hf/^{177}Hf$ initial	$\varepsilon_{Hf(t)}$	T_{DM1}(Ga)	T_{DM2}(Ga)
1	533	0.282534	0.000016	0.010433	0.000100	0.000268	0.000003	0.282531	3.21	1.00	1.30
2	533	0.282470	0.000016	0.009882	0.000224	0.000259	0.000009	0.282468	0.97	1.09	1.44
3	533	0.282522	0.000016	0.009012	0.000026	0.000213	0.000001	0.282519	2.80	1.01	1.32
4	533	0.282503	0.000014	0.007283	0.000092	0.000182	0.000002	0.282501	2.16	1.04	1.37
5	533	0.282422	0.000021	0.010369	0.000169	0.000264	0.000007	0.282419	-0.74	1.15	1.55
6	533	0.282521	0.000016	0.008874	0.000088	0.000213	0.000002	0.282519	2.77	1.01	1.33
7	533	0.282519	0.000015	0.008167	0.000071	0.000196	0.000001	0.282517	2.72	1.02	1.33
8	533	0.282505	0.000016	0.007526	0.000063	0.000175	0.000001	0.282503	2.24	1.03	1.36
9	533	0.282484	0.000015	0.007656	0.000042	0.000177	0.000001	0.282482	1.49	1.06	1.41
10	533	0.282488	0.000014	0.012351	0.000077	0.000270	0.000002	0.282485	1.59	1.06	1.40
11	533	0.282486	0.000014	0.005430	0.000041	0.000128	0.000001	0.282485	1.59	1.06	1.40
12	533	0.282387	0.000016	0.006344	0.000030	0.000151	0.000001	0.282386	-1.94	1.20	1.63
13	533	0.282494	0.000016	0.007373	0.000030	0.000167	0.000000	0.282492	1.84	1.05	1.39
14	533	0.282465	0.000017	0.007780	0.000033	0.000186	0.000002	0.282464	0.82	1.09	1.45
15	533	0.282505	0.000016	0.006970	0.000028	0.000179	0.000001	0.282503	2.23	1.03	1.36
16	533	0.282574	0.000017	0.007023	0.000028	0.000166	0.000000	0.282572	4.66	0.94	1.21
17	533	0.282479	0.000013	0.007163	0.000024	0.000165	0.000001	0.282478	1.33	1.07	1.42
18	533	0.282534	0.000015	0.006438	0.000049	0.000168	0.000002	0.282533	3.27	0.99	1.29
19	533	0.282503	0.000018	0.022040	0.000326	0.000646	0.000016	0.282496	1.98	1.05	1.38

Table S2. Major and trace element compositions of the hornblendites from Shitun village, Zhenghe County, South China.

.Sample	14WY-8-12	14WY-8-13	14WY-8-14	14WY-8-15
SiO_2	44.03	43.77	43.79	43.81
TiO_2	3.16	3.39	3.05	3.23
Al_2O_3	11.5	11.9	12.5	11.8
Fe_2O_3T	15.3	15.0	14.9	15.4
MnO	0.20	0.18	0.17	0.20
MgO	10.4	9.99	10.5	10.4
CaO	13.2	13.4	12.0	12.3
Na_2O	1.66	1.83	2.03	1.90
K_2O	0.39	0.33	0.58	0.66
P_2O_5	0.25	0.23	0.59	0.25
LOI	0.53	0.64	0.68	0.46
Total	100.03	99.97	99.87	99.54
V	455	455	425	466
Cr	421	426	381	430
Co	54.4	53.9	56.0	54.1
Ni	197	197	175	192
Ga	22.4	22.5	18.9	22.1
Rb	3.57	1.38	11.80	14.97
Sr	387	329	895	286
Y	33.3	34.7	36.0	32.6
Zr	196	231	205	195
Nb	13.5	14.9	17.2	14.1
Cs	1.47	0.21	0.57	0.49
Ba	62.3	55.7	272	181
La	20.6	22.1	23.2	21.6
Ce	55.0	61.6	60.3	56.4
Pr	8.49	9.08	8.79	8.61
Nd	39.1	41.3	40.0	39.1
Sm	9.27	9.92	9.55	9.28
Eu	2.75	2.93	3.64	2.95
Gd	8.68	9.22	9.04	8.58
Tb	1.34	1.41	1.43	1.33
Dy	7.49	7.92	7.97	7.25
Ho	1.38	1.48	1.52	1.37
Er	3.42	3.59	3.64	3.32
Tm	0.46	0.48	0.50	0.45
Yb	2.74	2.80	2.95	2.66
Lu	0.39	0.41	0.43	0.39
Hf	5.22	6.10	5.48	5.31
Ta	0.93	0.97	1.19	0.97
Pb	1.32	1.51	2.36	2.25
Th	0.94	1.36	2.57	1.69
U	0.38	0.52	0.79	0.54

Table S3. Whole rock Sr-Nd isotope compositions of the hornblendites.

Sample	Rb(ppm)	Sr(ppm)	$^{87}Rb/^{86}Sr$	$^{87}Sr/^{86}Sr$	2σ	Sm(ppm)	Nd (ppm)	$^{147}Sm/^{144}Nd$	$^{143}Nd/^{144}Nd$	2σ
14WY-8-12	3.57	387	0.026711	0.708661	0.000013	9.27	39.1	0.144176	0.512304	0.000006
14WY-8-13	1.38	329	0.012121	0.708166	0.000015	9.92	41.3	0.146009	0.512301	0.000004
14WY-8-14	11.8	895	0.038172	0.708426	0.000011	9.55	40.0	0.145383	0.512302	0.000008
14WY-8-15	15.0	286	0.151616	0.709514	0.000020	9.28	39.1	0.144441	0.512296	0.000009

Table S4. Whole rock Re-Os isotope compositions of the hornblendites.

Sample	Re(ppt)	2σ	Os(ppt)	2σ	$^{187}Re/^{188}Os$	2σ	$^{187}Os/^{188}Os$	2σ
14WY-8-12	1170	2.4	454	0.8	12.71	0.03	0.31612	0.00036
14WY-8-13	475	2.8	302	0.4	7.62	0.05	0.18313	0.00032
14WY-8-14	661	1.3	293	0.9	10.98	0.04	0.21730	0.00035
14WY-8-15	2669	2.5	313	0.8	42.98	0.11	0.47478	0.00044

Table S5. Representative chemical compositions of the hornblendes from the hornblendites.

Sample	Hbl_1	Hbl_2	Hbl_3	Hbl_4	Hbl_5	Hbl_6	Hbl_7	Hbl_8	Hbl_9	Hbl_{10}	Hbl_{11}	Hbl_{12}	Hbl_{13}	Hbl_{14}	Hbl_{15}	Hbl_{16}
SiO_2	41.75	44.83	44.96	44.84	45.04	44.56	44.3	44.63	44.86	44.99	49.25	43.69	44.53	44.78	44.38	40.76
TiO_2	0.53	1.16	1.31	2.02	1.95	1.97	0.82	2.03	1.66	1.77	0.35	3.17	1.87	2.23	1.02	0.85
Al_2O_3	15.5	11.6	11.4	11.2	11.2	11.3	12	11.3	11.2	11	7.32	11	11.2	11.2	11.9	15.9
Fe_2O_3T	15.9	14.5	14.6	14.7	14.8	14.7	14.4	15	14.5	14.9	13.2	15	14.7	14.6	15	16.3
MnO	0.19	0.19	0.26	0.22	0.19	0.16	0.19	0.2	0.21	0.2	0.19	0.22	0.16	0.18	0.2	0.21
MgO	9.47	11.4	11.2	11.1	11.1	10.8	10.8	11	11	11.2	13.6	10.4	10.9	10.8	10.7	8.8
CaO	11.8	11.8	11.9	11.7	11.6	11.5	11.8	11.5	11.6	11.8	12.1	12.3	12.3	12.1	11.8	11.6
Na_2O	2.25	1.95	1.88	1.9	1.67	1.92	1.85	1.98	1.83	1.86	1.13	1.83	1.91	1.84	1.9	2.38
K_2O	0.51	0.35	0.32	0.38	0.39	0.37	0.31	0.38	0.39	0.34	0.24	0.36	0.32	0.34	0.31	0.45
Total	97.9	97.78	97.83	98.06	97.94	97.28	96.47	98.02	97.25	98.06	97.38	97.97	97.89	98.07	97.21	97.25
TSi	6.169	6.574	6.604	6.581	6.595	6.592	6.593	6.55	6.627	6.601	7.145	6.509	6.593	6.607	6.569	6.087
TTi	1.831	1.426	1.396	1.419	1.405	1.408	1.407	1.45	1.373	1.399	0.855	1.491	1.407	1.393	1.431	1.913
CAl	0.866	0.578	0.577	0.517	0.526	0.56	0.696	0.503	0.575	0.502	0.396	0.439	0.546	0.553	0.643	0.883
CFe^{3+}	0.37	0.264	0.188	0.165	0.262	0.143	0.172	0.247	0.159	0.204	0.259	0	0	0	0.214	0.352
CTi	0.059	0.128	0.145	0.223	0.215	0.219	0.092	0.224	0.184	0.195	0.038	0.355	0.208	0.247	0.114	0.095
CMg	2.086	2.492	2.453	2.429	2.423	2.382	2.396	2.407	2.422	2.45	2.941	2.31	2.406	2.375	2.361	1.959
CFe^{2+}	1.595	1.515	1.606	1.639	1.55	1.675	1.62	1.594	1.632	1.625	1.342	1.869	1.82	1.801	1.643	1.684
CMn	0.024	0.024	0.032	0.027	0.024	0.02	0.024	0.025	0.026	0.025	0.023	0.028	0.02	0.022	0.025	0.027
BCa	1.868	1.854	1.873	1.84	1.82	1.823	1.882	1.808	1.836	1.855	1.881	1.963	1.951	1.913	1.871	1.856
BNa	0.132	0.146	0.127	0.16	0.18	0.177	0.118	0.192	0.164	0.145	0.119	0.037	0.049	0.087	0.129	0.144
ACa	0	0	0	0	0	0	0	0	0	0	0	0	0	0	0	0
ANa	0.513	0.409	0.408	0.381	0.294	0.373	0.415	0.372	0.36	0.384	0.199	0.492	0.499	0.439	0.417	0.545

Table S6. LA-ICP-MS trace element analyses of zircons from the hornblendite (14WY-8-15) and the calculated temperature depending on Ti content.

Spot	Ti	Y	Nb	La	Ce	Pr	Nd	Sm	Eu	Gd	Tb	Dy	Ho	Er	Tm	Yb	Lu	Hf	Th	U	ΣREE	T/°C
1	13.1	113	0.45		0.88	0.049	0.24	0.35	0.17	1.36	0.65	7.69	3.28	17.9	4.58	52.1	13.5	10431	22.1	53.5	102.7	752.4
2	9.98	92.0	0.29	0.044	0.40	0.023	0.16	0.31	0.24	1.22	0.54	6.57	2.68	13.9	3.42	41.5	11.2	9094	6.07	18.6	82.2	728.7
3	8.32	83.4	0.39	0.0060	1.08	0.0059	0.071	0.30	0.14	1.65	0.44	7.14	2.45	12.7	3.06	28.2	6.52	9275	15.1	47.9	63.8	713.2
4	194	164	0.45	0.0042	1.67	0.013	0.37	0.59	0.25	3.81	1.08	13.0	5.34	24.8	5.31	54.5	12.5	9600	31.2	80.5	123.3	*
5	9.04	86.6	0.32		0.81			0.18	0.15	1.35	0.48	6.85	2.58	13.9	3.29	35.7	8.93	9145	17.6	43.6	74.2	720.2
6	9.13	122	0.39	0.019	1.60	0.025	0.45	0.30	0.25	2.67	0.79	10.3	4.02	18.6	4.04	42.1	9.02	9226	35.7	96.5	94.2	721.0
7	90.7	111	0.40		0.69	0.037	0.042		0.22	2.41	0.76	8.73	3.48	17.1	3.96	41.8	10.5	8635	12.7	34.5	89.8	*
8	9.20	116	0.36	0.019	1.44	0.014	0.11	0.47	0.16	2.58	0.94	10.7	4.00	16.9	3.80	39.1	8.42	9155	27.8	69.5	88.7	721.7
9	8.22	129	0.31		1.54	0.023	0.17	0.73	0.21	2.97	0.93	11.0	4.11	19.3	4.46	42.0	9.24	9116	39.8	89.9	96.6	712.2
10	8.35	159	0.39	0.027	1.42	0.0069	0.58	0.52	0.39	3.72	1.05	14.4	5.27	23.8	5.11	49.5	10.9	9706	30.1	71.4	116.7	713.5
11	8.66	114	0.34		1.07			0.42	0.23	2.28	0.80	9.47	3.66	17.1	3.84	39.2	9.08	8730	20.3	43.6	87.1	716.6
12	8.98	247	0.36	0.0062	1.30	0.033	0.11	0.92	0.56	6.66	2.13	23.3	8.88	36.9	7.39	71.4	15.5	9338	28.8	55.7	175.1	719.6
13	8.82	79.0	0.26	0.0039	1.07	0.020	0.16	0.36	0.15	1.42	0.51	7.01	2.38	11.6	2.63	27.1	6.19	9071	19.5	55.3	60.6	718.1
14	8.44	99.1	0.33		1.55		0.070	0.15	0.21	2.34	0.71	8.19	3.05	14.7	3.15	32.9	6.94	8897	33.9	97.1	74.0	714.4
15	7.68	70.9	0.38	0.024	0.67	0.017		0.26	0.18	1.23	0.42	5.07	2.21	10.6	2.61	28.1	6.75	8670	11.0	25.7	58.2	706.6
16	6.79	101	0.26	0.0000	0.55	0.012	0.26	0.36	0.24	1.92	0.66	8.65	3.20	15.2	3.40	36.7	8.56	8307	9.23	24.6	79.7	696.5
17	8.85	160	0.50		1.83		0.22	0.80	0.28	3.59	1.15	13.5	5.05	24.5	5.16	52.7	11.4	9096	34.5	92.5	120.1	718.4
18	5.33	74.8	0.32	0.0099	0.49	0.010		0.13	0.14	1.39	0.33	4.99	2.22	11.8	2.80	33.3	8.82	9042	9.55	22.9	66.4	677.3
19	8.11	234	0.46		1.94	0.0077	0.36	0.73	0.30	4.42	1.40	17.9	7.73	36.7	7.98	86.9	21.3	9980	77.8	105	187.7	711.0

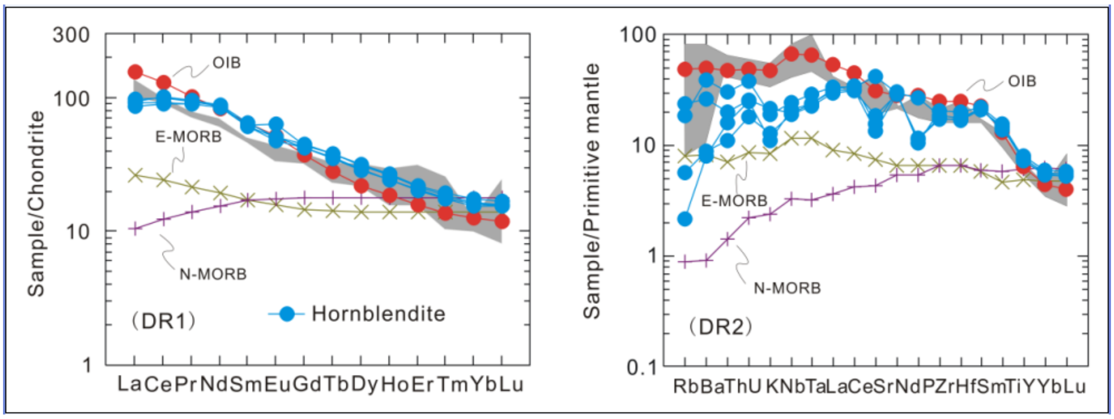

Fig.DR1 Chondrite normalized REE distributions for the hornblendites from the Cathaysia Block of South China.

Fig.DR2 Primitive mantle normalized spidergram of the hornblendites. OIB, Chondrite and primitive mantle-normalieze values are from Sun and McDonough [50].

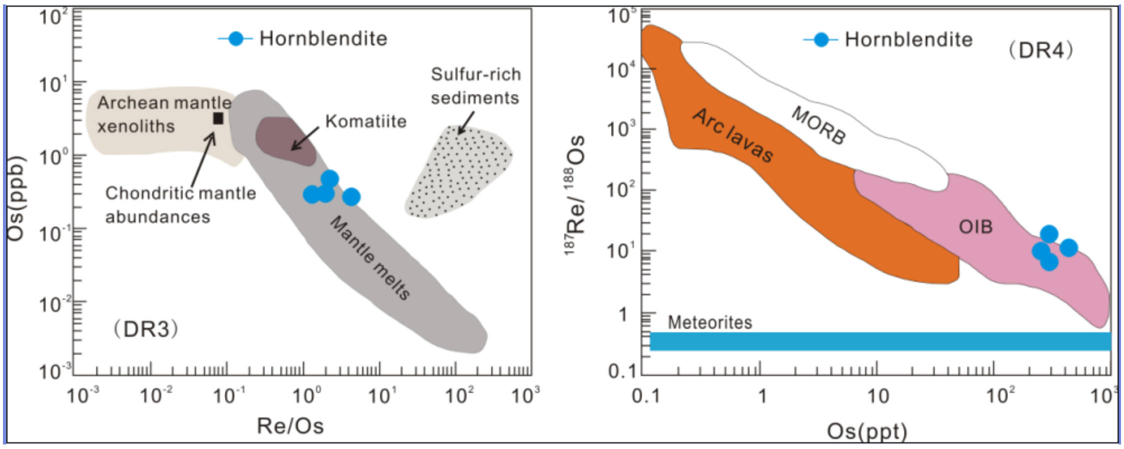

Fig.DR3 Os-Re/Os plots for discriminating different sources for the hornblendites. Fig.DR4 $^{187}Re/^{188}Os$ ratio vs. Os concentrations for the hornblendites. Arc lavas, MORB and OIB are from Alves *et al.*[51].

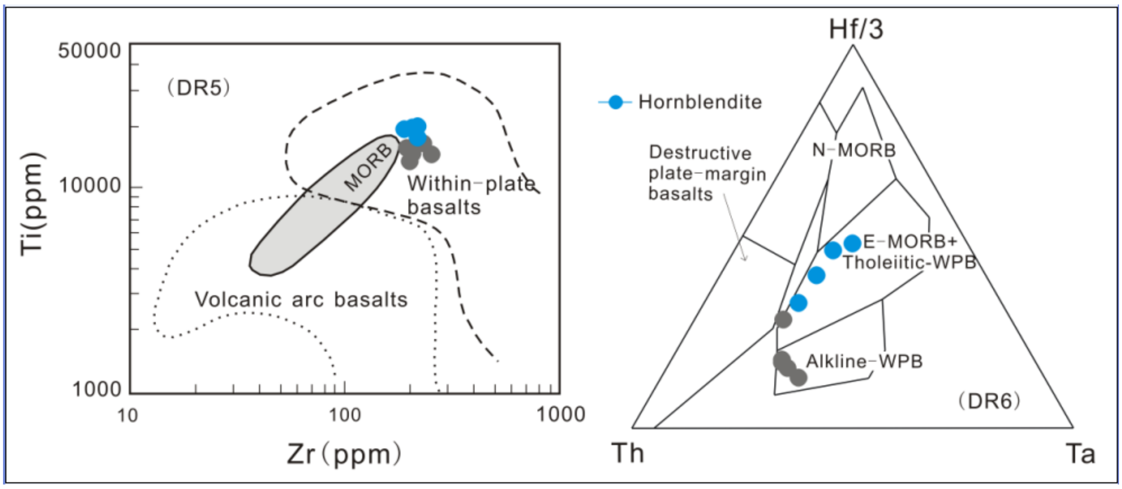

Fig.DR5-6. Tectonic discriminant diagrams for the hornblendites from the Cathaysia Block. (DR5)Ti–Zr diagram[52, 53]; (DR6) Th–Ta–Hf/3 diagram[54].

50. Sun, S.S., McDonough, W.F., 1989. Chemical and isotopic systematics of oceanicbasalts: implications for mantle composition and processes. In: Saunders, A.D.,Norry, N.J. (Eds.), Magmatism in the ocean basins. Geol. Soc. Lond. (Spec. Publ.)42, 313-345.
51. Alves, S., Schiano, P., Capmas, F., Allergre, C.J., 2002. Osmium isotope binary mixing arrays in arc volcanism. Earth and Planetary Science Letters 198, 355-369.
52. Pearce, J.A., Thorpe, R.S., 1982. Andesites: Trace Element Characteristics of Lavasfrom Plate Boundaries. Wiley and Sons, New York, pp. 525-548.
53. Condie, K.C., 1989. Plate Tectonics and Crustal Evolution. Pergamon Press, Oxford,476 pp.
54. Wood, D.A., 1980. The application of a Th–Hf–Ta diagram to problems of tectonomagmatic classification and to establishing the nature of crust contamination of basaltic lavas of the British Tertiary volcanic province. Earth and Planetary Science Letters 50, 11-30.
